# Presence of Cardiometabolic Risk Factors Is Not Associated with Microalbuminuria in 14-to-20-Years Old Slovak Adolescents: A Cross-Sectional, Population Study

**DOI:** 10.1371/journal.pone.0129311

**Published:** 2015-06-05

**Authors:** Radana Gurecká, Ivana Koborová, Jozef Šebek, Katarína Šebeková

**Affiliations:** 1 Institute of Molecular BioMedicine, Faculty of Medicine, Comenius University, Bratislava, Slovakia; 2 Institute of Technology, Slovak Academy of Sciences, Bratislava, Slovakia; Sickle Cell Unit, JAMAICA

## Abstract

**Introduction:**

In adults, microalbuminuria indicates generalized endothelial dysfunction, and is an independent risk factor for cardiovascular and all cause mortality. Slovak adults present one of the highest cardiovascular mortality rates in Europe. Thus Slovak adolescents are on a high-risk to develop cardiovascular afflictions early, and screening for microalbuminuria might be useful in early assessment of their cardiovascular risk. We aimed to study the prevalence of microalbuminuria in Slovak adolescents, and the association of urinary albumin-to-creatinine ratio (ACR) to cardiovascular risk factors.

**Subjects and methods:**

Anthropometric data, blood pressure, blood count, glucose homeostasis, lipid profile, renal function, inflammatory status, concentrations of homocysteine and uric acid were determined and associated with ACR in 2 666 adolescents (49.4% boys, 51.6% girls) aged 14-to-20 years. Microalbuminuria was classified as ACR 2.5–25.0 mg/mmol in boys and 3.5–35.0 mg/mmol in girls.

**Results:**

Prevalence of microalbuminuria in both genders reached 3.3%, and did not differ significantly between lean and centrally obese subjects. Girls presented higher ACR than boys (normoalbuminuric: 0.6±0.5 mg/mmol vs. 0.5±0.4 mg/mmol, p>0.001; microalbuminuric: 9.3±7.3 mg/mmol vs. 5.0±3.8 mg/mmol; p>0.001). Microalbuminuric adolescents and those presenting normoalbuminuria within the upper ACR quartile were slimmer than their normoalbuminuric counterparts or adolescents with normoalbuminuria within the lower quartile, respectively. No association between microalbuminuria and cardiovascular risk markers was revealed.

**Conclusion:**

Results obtained in this study do not support our assumption that ACR associates with cardiometabolic risk factors in apparently healthy adolescents. Follow-up studies until adulthood are needed to estimate the potential cardiometabolic risk of apparently healthy microalbuminuric adolescents.

## Introduction

Glomerular capillaries generally conserve 99.9% of proteins, thus increased urinary albumin excretion (UAE) rate reflects injury to both renal and systemic vascular beds and indicates generalized endothelial dysfunction [1, 2]. The term microalbuminuria (MA) indicates the presence of a small amount of albumin (a concentration below the detection threshold of a standard urine dipstick test) in the urine.

In adults MA indicates either presence of renal or cardiovascular (CV) disease or an enhanced risk for their development [3–7]; and is considered the first clinical sign of obesity-associated nephropathy, particularly in the presence of hypertension [8].

Prevalence of MA in general population of adolescents reaches 4%-to-16% [9–15], thus is higher than in normotensive non-diabetic adults, ranging between 3%-to-8% [7, 16, 17]. However, healthy lean adolescents present, paradoxically, significantly higher prevalence of MA than their overweight and obese counterparts [10, 12, 14, 15, 18–21]. Unlike in adults, in adolescents correlation between MA and cardiovascular risk factors has not been clearly proved yet. Only two studies reported a significant association between MA and CV risk factors in apparently healthy adolescents: Rademacher et al. [22] revealed a positive relationship between UAE rate and fasting insulin; while in black males (but white males and black or white females) UAE rate correlated directly with systolic blood pressure [23]. In a large study of Nguyen et al. [10], MA was associated with impaired fasting glucose, insulin resistance, and hypertension only in overweight adolescents.

Prevalence of overweight and obesity is alarmingly increasing among Slovak youth [24]. The onset of obesity in childhood and adolescence may manifest in consequences such as hypertension, insulin resistance, metabolic syndrome, type 2 diabetes mellitus, and other diseases already in youth, and increases the likelihood of premature cardiometabolic morbidity and mortality in adulthood [25–27]. Among EU-27 countries Slovakia holds the 5^th^ top position in standardized death rate from CV and the 2^nd^ highest position in that from ischemic heart disease [28, 29]. Thus, Slovak adolescents are on high risk to develop CV disease.

We hypothesize that if MA is an indicator of early vascular damage in the adolescents, ACR should associate with cardiometabolic risk factors. Considering the obese children at particularly high risk of CV outcomes, we aim to study whether central obesity modifies the association between ACR and cardiometabolic risk factors. The prevalence of MA (determined as ACR in the first voided morning sample), and the associations of ACR to cardiometabolic risk factors (anthropometric characteristics, blood pressure values, biomarkers reflecting glucose homeostasis, lipid profile, renal function, low grade inflammation; uric acid and homocysteine) was assessed in 2 666 apparently healthy adolescents. We used principal component analysis for statistical evaluation, assuming that multivariate analysis dealing with dummy variables representing an eigenvector in multidimensional space for each individual studied could reveal associations not discovered in standard multivariate approaches handling series of variables *per se* in parallel.

## Subjects and methods

The “Respect for Health” study has been launched in 2011 in cooperation of two local health authorities—Department of Health of Bratislava Self-governing Region and the Regional Public Health Authority of the Slovak Republic in Bratislava. This cross-sectional study aims to characterize cardiometabolic health status of students attending state-governed secondary schools in Bratislava region and to establish an effective preventive program for early cardio- and nephro-protective regimens. Data were collected from November 2011 to December 2012: anthropometric and blood pressure (BP) measurements were performed directly at high schools by educated, trained staff; blood and urine analyses in a central laboratory.

Decision to participate was on a voluntary basis. A signed informed consent was obtained from the adult participants. The minors provided verbal assent, and an informed consent signed by their parents/guardians. Study was conducted according to the Declaration of Helsinki, after the approval of the protocol by the Ethics Committee of Bratislava Self-Governing Region.

### Subjects

In school-year 2011/2012, 19 172 students were enrolled in high schools in Bratislava region. Four thousand four hundred (22.9%) adolescents aged 12-to-23 years underwent anthropometric measurements. From among them 2 960 (67.3%) provided blood and urine samples. After exclusion of students aged <14 years (in whom Tanner stage significantly and independently affects UAE rate [12]); those aged >20 years (due to small number, and considerable prevalence of boys) (n = 30); subjects of non-Middle European descent (n = 28), and those in whom complete blood and urine analyses data were not available (n = 224), subjects presenting fasting serum glucose ≥6.9 mmol/l (n = 7, albuminuria in them might have reflected diabetic nephropathy), and those with ACR in proteinuric range (n = 5, albuminuria might have reflected primary renal disease); data from 2 666 adolescents (49.4% boys, 51.6% girls) aged 14-to-20-years were available for final evaluation. This accounts for 90.1% of students who underwent anthropometric measurements and provided biological material. A representative sample for 14-to-20-year-old enrolled students, with 95% confidence interval and 80% power, was calculated as 1250 boys and 1260 girls.

### Measurements

Anthropometric measurements were performed directly at high schools by trained working groups comprising students from Slovak Medical University, supervised by PhD students from Slovak Medical University and Comenius University, or employees of the Regional Public Health Authority. Waist circumference was taken with the subjects standing upright, with relaxed shoulders and facing the investigator, with non-elastic tape at midway between the lowest rib and the iliac crest. Height was measured by portable stadiometer. Body weight was assessed using digital scales (Omron BF510, Kyoto, Japan) equipped for determination of total body fat percentage employing bioimpedance method. Blood pressure was measured by digital BP monitor (Omron M-6 COMFORT, Kyoto, Japan) on the right arm, after comfortably seated subject relaxed for at least 10 minutes. An average of the last 2 measurements out of 3 was taken as the resultant value. Body mass index (BMI) and waist-to-height ratio were calculated. Subjects displaying waist/height >0.50 were classified as centrally obese [30]. A new body shape index (ABSI) [31] was calculated using scaling coefficients derived from our cohort, separately for girls and boys. For this calculation waist circumference measured according to the NHANES protocol was used [32]. Coefficients of regressing log waist circumference upon log weight and log height were calculated for both genders separately and rounded to ratio of small integers [31].

### Blood and urine analyses

Blood and urine samples were collected at 5 health centers located in Bratislava region, and thereafter transferred by blood curriers to the central laboratory for analyses. Blood was withdrawn in the morning hours (7.00–9.00 a.m.) after overnight fasting. Blood count and concentrations of glucose, insulin, albumin, creatinine, urea, uric acid, total and high-density lipoprotein (HDL)-cholesterol, triacylglycerols (TAG), and high-sensitive C-reactive protein (hsCRP) were measured using standard laboratory methods (Advia 2400 analyzer, Siemens). Spot urine samples from the first morning void at home were collected, and analyzed for creatinine (kinetic Jaffe’s reaction), albumin concentration (immunoturbidimetrically) and specific gravity. Calculations: low-density lipoprotein cholesterol (LDL-C) via the Friedewald equation; insulin sensitivity using Quantitative Insulin Sensitivity Check Index (QUICKI) [33]; atherogenic index of plasma (AIP) as log(TAG/HDL-C) [34]; estimated glomerular filtration rate (eGRF) via Schwartz (in subjects aged ≤18 years) and MDRD (in 19–20 year-olds) formulas [35, 36]. ACR of 2.5–25.0 mg/mmol in boys and 3.5–35.0 mg/mmol in girls were classified as MA, lower values as normoalbuminuria (NA), and higher as overt proteinuria [37]. Although derived from population studies in adults, these cut-offs are used also in the pediatric populations.

### Statistical analyses

The primary outcomes for the analysis were MA as categorical, and ACR as continuous variable. Due to different ACR cut-off values males and females were evaluated separately. Data not fitting to normal distribution were logarithmically transformed prior to statistical evaluation, but for better understanding means±SD are presented, if not indicated differently. Multivariate analyses—Principal component analysis (PCA) and Orthogonal projections to latent structures discriminant analysis (OPLS-DA) were performed to identify variables contributing to between group separation (e.g. NA vs. MA), using Simca v.13 software (Umetrics, Umea, Sweden). Variables with Variable of importance for the projection (VIP) values >1 were considered important contributors to between group separation, those with VIP <0.5 unimportant. VIP ranging between 0.5-to-1 is referred to as a “grey interval”, importance of the variable depends on the sample size. Two-sided Student's T-test was used to compare 2 groups—NA vs. MA, lower vs. upper quartile within NA range, lean vs. centrally obese. Categorical data were compared using chi-square test (with Yates’s correction, if appropriate). SPSS statistical software (v.16 for Windows, SPSS, Chicago, Illinois) was used, with a significance set at p<0.05.

## Results

Cohort characteristics are given in Table 1. Girls and boys differed significantly in all variables except for age, insulin sensitivity and TAG levels.

**Table 1:** Anthropometric and biochemical characteristics of the subjects.

	Boys	Girls
N	1 316	1 350
Age (years)	16.8 ± 1.3	16.8 ± 1.2
Height (cm)	179.0 ± 6.8	165.7 ± 6.2***
Weight (kg)	74.3 ± 13.8	60.3 ± 10.3***
Waist cf. (cm)	79.5 ± 9.1	71.6 ± 7.7***
Waist/height	0.44 ± 0.05	0.43 ± 0.05***
BMI (kg/m2)	23.1 ± 3.8	21.9 ± 3.4***
ABSI	0.0673 ± 0.0038	0.0979 ± 0.0064***
Total body fat (%)	*17*.*5± 7*.*3*	*30*.*3± 6*.*9* ***
SBP (mm Hg)	123 ± 12	107 ± 9***
DBP (mm Hg)	73 ± 8	70 ± 8***
PP (mm Hg)	50 ± 10	37 ± 7***
MAP (mm Hg)	89 ± 8	83 ± 8***
Albumin (g/l)	48.5 ± 2.2	47.2 ± 2.4***
FPG (mmol/l)	4.9 ± 0.4	4.7 ± 0.4***
FPI (mIU/l)	*11*.*0 ± 7*.*6*	*11*.*1 ± 6*.*1* *
QUICKI	0.344 ± 0.027	0.343 ± 0.024
Cholesterol (mmol/l)	3.83 ± 0.70	4.24 ± 0.75***
HDL-C (mmol/l)	1.25 ± 0.23	1.51 ± 0.30***
LDL-C (mmol/l)	2.18 ± 0.59	2.33 ± 0.60***
TAG (mmol/l)	*0*.*88 ± 0*.*46*	*0*.*89 ± 0*.*39*
AIP	-0.44 ± 0.52	-0.60 ± 0.46***
Uric acid (μmol/l)	355 ± 60	259 ± 50***
hsCRP (mg/l)	*1*.*07 ± 2*.*08*	*1*.*41 ± 3*.*12* *
Hcy (μmol/l)	*12*.*1 ± 6*.*1*	*9*.*8 ± 3*.*2* ***
Urea (mmol/l)	4.47 ± 1.06	3.73 ± 0.89***
Creatinine (μmol/l)	76 ± 13	61 ± 8***
eGFR (ml/s/1.73m2)	1.70 ± 0.25	1.54 ± 0.22***
ACR (mg/mmol)	*0*.*61 ± 1*.*12*	*0*.*90 ± 2*.*11* ***
Urine density (kg/m3)	1021 ± 6	1020 ± 7***
WBC (x.109/l)	6.4 ± 1.4	6.9 ± 1.8***
RBC (x.1012/l)	5.1 ± 0.3	4.5 ± 0.3***
Platelets (x.109/l)	248 ± 49	275 ± 58***

cf.: circumference; BMI: body mass index; ABSI: a new body shape index; SBP: systolic blood pressure; DBP: diastolic blood pressure; PP: pulse pressure; MAP: mean arterial pressure; FPG: fasting serum glucose; FPI: fasting serum insulin; IU: international units; QUICKI: quantitative insulin sensitivity check index; HDL: high density lipoprotein; C: cholesterol; LDL: low density lipoprotein; TAG: triacylglycerols; AIP: atherogenic index of plasma; hsCRP: high sensitive C-reactive protein; eGFR: estimated glomerular filtration rate; WBC: white blood cells count; PBC: red blood cells count; NS: not significant; *italics*: *not normally distributed data evaluated statistically after logarithmic transformation*; data presented as mean ± SD;

* p<0.05,

** p<0.01,

***p<0.001 vs. boys

### MA prevalence and level—between gender comparisons

Boys, regardless whether normoalbuminuric or microalbuminuric (Tables 1 and 2), presented lower ACR (p>0.001, all) if compared with girls (Tables 1 and 3). Prevalence of MA reached 3.3% in both genders (Tables 2 and 3).

**Table 2:** Characteristics of boys presenting normo- and microalbuminuria, and albumin-to-creatinine ratio (ACR) in the upper and lower quartiles of normoalbuminuric range.

	Normoalbuminuria	Microalbuminuria	Lower quartile of NA	Upper quartile of NA
	ACR < 2.5 mg/mmol	ACR = 2.5–25.0 mg/mmol	ACR ≤ 0.23 mg/mmol	ACR ≥ 0.58 mg/mmol
N	1 273 (96.7%)	43 (3.3%)	296	327
Age (years)	16.8 ± 1.3	16.7 ± 1.4	17.0 ± 1.2	16.7 ± 1.3^††^
Height (cm)	179.0 ± 6.8	179.4 ± 6.2	180.1 ± 6.5	179.0 ± 6.9^†^
Weight (kg)	74.4 ± 13.9	70.2 ± 10.9*	76.5 ± 13.4	72.3 ± 13.3^†††^
Waist cf. (cm)	79.6 ± 9.1	75.8 ± 6.4**	80.3 ± 8.8	75.2 ± 8.8^††^
Waist/height	0.45 ± 0.05	0.42 ± 0.04**	0.45 ± 0.05	0.44 ± 0.05^†^
BMI (kg/m2)	23.2 ± 3.8	21.8 ± 3.0*	23.5 ± 3.8	22.5 ± 3.7^††^
ABSI	0.0673 ± 0.0042	0.0678 ± 0.0040	0.0669 ± 0.0035	0.0676 ± 0.0036^†^
Total body fat (%)	*17*.*5 ± 7*.*3*	*15*.*1 ± 5*.*7* *	*18*.*1 ± 7*.*1*	*16*.*3 ± 7*.*3* ^††^
SBP (mm Hg)	123 ± 12	121 ± 12	124 ± 12	121 ± 11
DBP (mm Hg)	73 ± 8	73 ± 9	73 ± 8	72 ± 8
PP (mm Hg)	50 ± 10	47 ± 8	51 ± 10	49 ± 9^††^
MAP (mm Hg)	89 ± 8	89 ± 9	89 ± 9	89 ± 8
Albumin (g/l)	48.5 ± 2.2	48.6 ± 2.6	48.2 ± 2.1	48.6 ± 2.3
FPG (mmol/l)	4.9 ± 0.4	4.9 ± 0.4	4.9 ± 0.4	4.9 ± 0.4
FPI (mIU/l)	*11*.*1 ± 7*.*5*	*10*.*9 ± 9*.*9*	*11*.*1 ± 6*.*8*	*11*.*1 ± 7*.*8*
QUICKI	0.344±0.027	0.349.032	0.342±0.027	0.344.027
Cholesterol (mmol/l)	3.82 ± 0.70	3.87 ± 0.86	3.88 ± 0.69	3.76 ± 0.74^†^
HDL-C (mmol/l)	1.25 ± 0.23	1.28 ± 0.22	1.26 ± 0.22	1.26 ± 0.23
LDL-C (mmol/l)	2.18 ± 0.58	2.21± 0.71	2.20 ± 0.57	2.12± 0.60
TAG (mmol/l)	*0*.*88 ± 0*.*47*	*0*.*84 ± 0*.*27*	*0*.*92 ± 0*.*49*	*0*.*84 ± 0*.*44* ^††^
AIP	-0.44 ± 0.53	-0.46 ± 0.36	-0.40 ± 0.51	-0.49 ± 0.51^†^
Uric acid (μmol/l)	355 ± 59	351 ± 69	363 ± 61	346 ± 60^††^
hsCRP (mg/l)	*1*.*07 ± 2*.*07*	*1*.*02 ± 2*.*37*	*1*.*05 ± 1*.*68*	*0*.*94 ± 2*.*08* ^†^
Hcy (μmol/l)	*12*.*2 ± 6*.*2*	*11*.*0 ± 3*.*5*	*12*.*0 ± 5*.*8*	*12*.*0 ± 6*.*5*
Urea (mmol/l)	4.45 ± 1.05	4.90 ± 1.24**	4.57 ± 1.02	4.52 ± 1.20
Creatinine (μmol/l)	77 ± 13	75 ± 9	79 ± 13	74 ± 12^†††^
eGFR (ml/s/1.73m2)	1.70 ± 0.25	1.76± 0.21	1.65 ± 0.25	1.76± 0.25^†††^
ACR	*0*.*46 ± 0*.*38*	*4*.*98± 3*.*82* ***	*0*.*16 ± 0*.*05*	*1*.*54± 1*.*96* ^†††^
Urine density (kg/m3)	1021 ± 7	1023 ± 6*	1019 ± 7	1022 ± 7^†††^
WBC (x.109/l)	6.4 ± 1.4	6.3 ± 1.4	6.4 ± 1.4	6.3 ± 1.4
RBC (x.1012/l)	5.1 ± 0.3	5.1 ± 0.2	5.2 ± 0.3	5.1 ± 0.3
Platelets (x.109/l)	248 ± 49	250 ± 50	248 ± 50	248 ± 49

cf.: circumference; BMI: body mass index; ABSI: a new body shape index; SBP: systolic blood pressure; DBP: diastolic blood pressure; PP: pulse pressure; MAP: mean arterial pressure; FPG: fasting serum glucose; FPI: fasting serum insulin; IU: international units; QUICKI: quantitative insulin sensitivity check index; HDL: high density lipoprotein; C: cholesterol; LDL: low density lipoprotein; TAG: triacylglycerols; AIP: atherogenic index of plasma; hsCRP: high sensitive C-reactive protein; eGFR: estimated glomerular filtration rate; WBC: white blood cells count; PBC: red blood cells count; NS: not significant; *italics*: *not normally distributed data evaluated statistically after logarithmic transformation*; data presented as mean ± SD;

* p<0.05,

** p<0.01,

***p<0.001 vs. normoalbumiuria;

^†^ p<0.05,

^††^ p<0.01,

^†††^p<0.001 vs. lower quartile

**Table 3:** Characteristics of girls presenting normo- and microalbuminuria, and albumin-to-creatinine ratio (ACR) in the upper and lower quartiles of normoalbuminuric range.

	Normoalbuminuria	Microalbuminuria	Lower quartile of NA	Upper quartile of NA
	ACR < 3.5 mg/mmol	ACR = 3.5–35.0 mg/mmol	ACR ≤ 0.29 mg/mmol	ACR ≥ 0.76 mg/mmol
N	1 305 (96.7%)	45 (3.3%)	314	333
Age (years)	16.9 ± 1.2	16.5 ± 1.1	16.9 ± 1.1	16.9 ± 1.3
Height (cm)	165.7 ± 6.2	166.4 ± 6.7	166.5 ± 6.1	166.2 ± 6.4
Weight (kg)	60.3 ± 10.2	58.1 ± 10.5	61.9 ± 10.5	58.9 ± 10.6^†††^
Waist cf. (cm)	71.7 ± 7.7	70.8 ± 8.8	72.7 ± 8.0	70.7 ± 7.7^††^
Waist/height	0.43 ± 0.05	0.43 ± 0.05	0.44 ± 0.05	0.43 ± 0.05^††^
BMI (kg/m2)	22.0 ± 3.4	21.0 ± 3.5	22.3 ± 3.5	21.3 ± 3.5^†††^
ABSI	0.0979 ± 0.0064	0.0978 ± 0.0071	0.0980 ± 0.0062	0.0989 ± 0.0067
Total body fat (%)	*30*.*4± 6*.*9*	*27*.*6± 8*.*3* *	*31*.*1± 7*.*0*	*29*.*2± 6*.*9* ^†††^
SBP (mm Hg)	107 ± 9	109 ± 9	107 ± 10	108 ± 10
DBP (mm Hg)	70 ± 8	71 ± 7	71 ± 7	71 ± 8
PP (mm Hg)	37 ± 7	387± 7	37 ± 7	37± 7
MAP (mm Hg)	83 ± 8	84 ± 7	83 ± 8	83 ± 8
Albumin (g/l)	47.2 ± 2.3	47.9 ± 3.1	46.8 ± 2.3	47.3 ± 2.4^††^
FPG (mmol/l)	4.7 ± 0.4	4.7 ± 0.4	4.6 ± 0.4	4.7 ± 0.4^†^
FPI (mIU/l)	*11*.*1 ± 6*.*1*	*10*.*6 ± 5*.*5*	*11*.*3 ± 7*.*1*	*11*.*1 ± 6*.*0*
QUICKI	0.343 ± 0.024	0.347 ± 0.029	0.344 ± 0.025	0.343 ± 0.025
Cholesterol (mmol/l)	4.25 ± 0.75	4.16 ± 0.72	4.30 ± 0.74	4.16 ± 0.75^†^
HDL-C (mmol/l)	1.51 ± 0.30	1.50 ± 0.25	1.51 ± 0.30	1.52 ± 0.29
LDL-C (mmol/l)	2.33 ± 0.60	2.28 ± 0.61	2.37 ± 0.59	2.25 ± 0.61^†^
TAG (mmol/l)	*0*.*89 ± 0*.*40*	*0*.*83 ± 0*.*31*	*0*.*92 ± 0*.*40*	*0*.*85 ± 0*.*37* ^††^
AIP	-0.60 ± 0.46	-0.64 ± 0.45	-0.55 ± 0.45	-0.65 ± 0.45
Uric acid (μmol/l)	259 ± 51	263 ± 42	267 ± 49	252 ± 50^†††^
hsCRP (mg/l)	*1*.*39 ± 2*.*96*	*1*.*99 ± 6*.*13*	*1*.*42 ± 2*.*74*	*1*.*39 ± 3*.*22*
Hcy (μmol/l)	*9*.*8 ± 3*.*1*	*10*.*5 ± 5*.*1*	*9*.*8 ± 3*.*2*	*9*.*8 ± 3*.*2*
Urea (mmol/l)	3.73 ± 0.90	3.72 ± 0.76	3.78 ± 0.92	3.72 ± 0.89
Creatinine (μmol/l)	61 ± 8	62 ± 7	63 ± 8	60 ± 8^†††^
eGFR (ml/s/1.73m2)	1.55 ± 0.22	1.51 ± 0.20	1.50 ± 0.21	1.57 ± 0.24^†††^
ACR	*0*.*61 ± 0*.*54*	*9*.*28 ± 7*.*30* ***	*0*.*20 ± 0*.*06*	*2*.*46 ± 3*.*84* ^†††^
Urine density (kg/m3)	1020 ± 7	1018 ± 7	1018 ± 7	1021 ± 7^††^
WBC (x.109/l)	6.9 ± 1.8	7.0 ± 1.9	6.9 ± 1.6	6.8 ± 1.7
RBC (x.1012/l)	4.5 ± 0.3	4.6 ± 0.3	4.6 ± 0.3	4.5 ± 0.3
Platelets (x.109/l)	275 ± 58	271 ± 44	278 ± 57	273 ± 56

cf.: circumference; BMI: body mass index; ABSI: a new body shape index; SBP: systolic blood pressure; DBP: diastolic blood pressure; PP: pulse pressure; MAP: mean arterial pressure; FPG: fasting serum glucose; FPI: fasting serum insulin; IU: international units; QUICKI: quantitative insulin sensitivity check index; HDL: high density lipoprotein; C: cholesterol; LDL: low density lipoprotein; TAG: triacylglycerols; AIP: atherogenic index of plasma; hsCRP: high sensitive C-reactive protein; eGFR: estimated glomerular filtration rate; WBC: white blood cells count; PBC: red blood cells count; NS: not significant; *italics*: *not normally distributed data evaluated statistically after logarithmic transformation*; data presented as mean ± SD;

* p<0.05,

** p<0.01,

***p<0.001 vs. normoalbumiuria;

^†^ p<0.05,

^††^ p<0.01,

^†††^p<0.001 vs. lower quartile

Mean ACR among microalbuminuric subjects was low (Tables 2 and 3), in both genders within the 1^st^ quartile of the respective microalbuminuric range. Among microalbuminuric boys 91% presented ACR values within the lower, and none of them within the upper quartile. 71% of microalbuminuric girls presented ACR values within the 1^st^ and 7% within the 4^th^ quartile.

### Association between microalbuminuria and cardiometabolic risk factors

#### Boys

**Fig 1:**
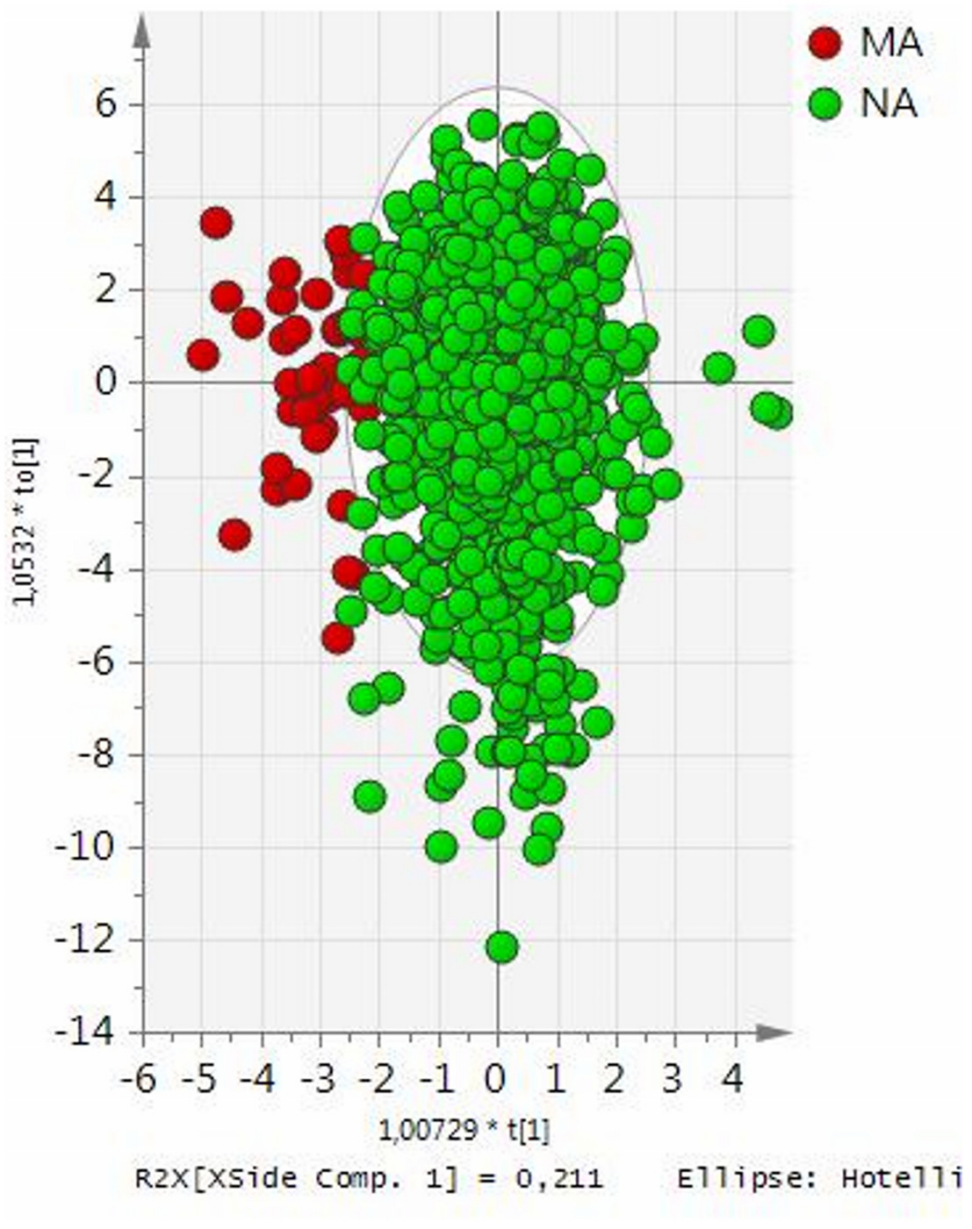
Score scatter plot from OPLS-DA model of normoalbuminuric (NA, green circles) and microalbuminuric (MA, red circles) boys. Scores are orthogonal (= completely independent from each other), representing new variables summarizing the input of all determined variables (herein the morphometric and biochemical variables) so that one score vector corresponds to one subject, having its own score vector. Observations situated far outside Hotelling’s T2 tolerance ellipse are outliers. Model reveals partial overlapping of NA and MA subjects (separation in direction of x-axis). Separation in direction of y-axis represents within group variability.

**Fig 2:**
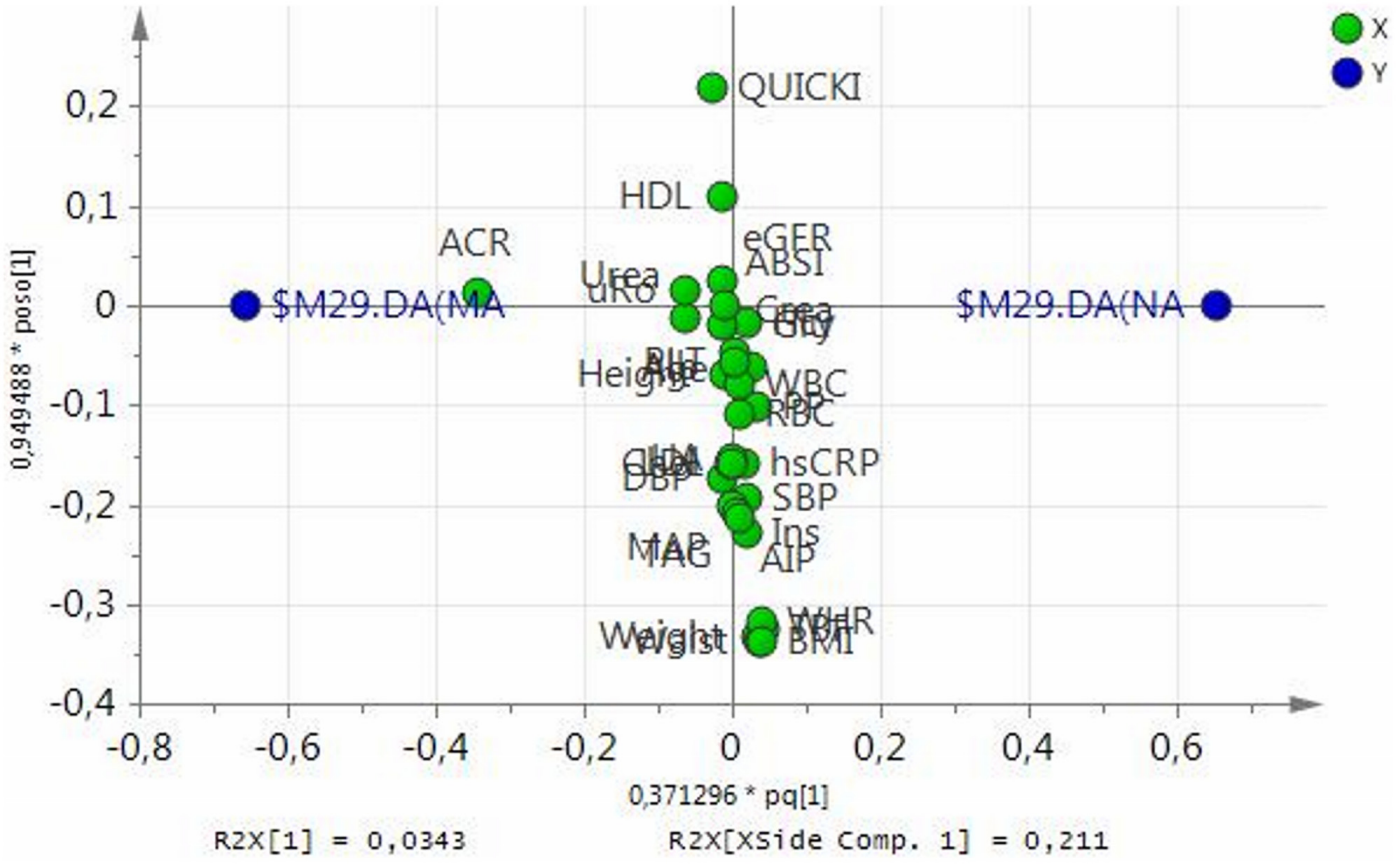
Loading scatter plot from OPLS-DA model of normoalbuminuric (NA) and microalbuminuric (MA) boys. Dummy variables (blue circles) characterize the respective 2 groups categorized according to absence (DANA) and presence (DAMA) of microalbuminuria. Albumin-to-creatinine ratio (ACR) adjacent to dummy variable DAMA represents the most significant component with discriminatory power determining the separation between the groups. Microalbuminuric boys also tend to present higher urine density and serum urea levels, and low total body fat percentage, body mass index, waist-to-height ratio, waist circumference and body weight (far right to dummy DAMA variable). Variables positioned near to intersect are similar in NA and MA cohorts thus do not contribute to between groups separation.

**Fig 3:**
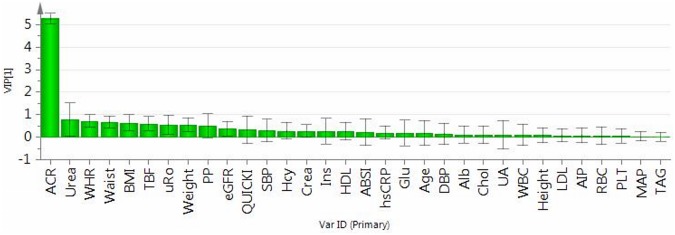
Plot of variables of importance contributing to between group separation among normoalbuminuric and microalbuminuric boys. Plot of variables importance for the projection (VIP) summarizes the importance of the variables both to explain X and to correlate to dummy variables (Figs 1 and 2). VIP values >1 indicate „important”X variables, <0.5 “unimportant” X variables, in the “grey interval” (0.5-to-1) the importance depends on the sample size. This plot confirms the OPLS-DA loadings scatter plot (Fig 2), showing that the variables adjacent to the origin in the former plot do not contribute to between group separation significantly. Abbreviations used in Figs 2 and 3: ACR: urinary albumin-to-creatinine ratio; WHR: waist-to-height ratio; waist: waist circumference; BMI: body mass index; uRo: urine density; weight: body weight; PP: pulse pressure; eGFR: estimated glomerular filtration rate; QUICKI: quantitative insulin check index; SBP: systolic blood pressure; Hcy: homocysteine; Crea: serum creatinine; Ins: fasting serum insulin; HDL: high density lipoprotein cholesterol; ABSI: a new body shape index; hsCRP: high sensitivity C-reactive protein; Glu: fasting serum glucose; DBP: diastolic blood pressure; Alb: serum albumin; Chol: cholesterol; UA: serum uric acid; WBC: white blood cells count; LDL: low density lipoprotein cholesterol; AIP: atherogenic index of plasma; RBC: red blood cells count; Plt: platelet count; MAP: mean arterial pressure; TAG: triacylglycerols.

PCA: The scores scatter plot showed that >95% of scores were situated within the Hotelling’s T2 tolerance ellipse, without major outliers, apparent groupings or similarities. Five component model was calculated, explaining 55% of variation, but with poor predictability (21%). The loadings scatter plot suggested that the model does not explain ACR well. OPLS-DA model revealed apparent separation between the NA and MA groups (Fig 1) but variation explained by the model (R^2^ = 30%) and its ability to predict new data (Q^2^ = 28%) were poor. ACR appeared as the major variable contributing to the separation between the groups (Fig 2), with variable of importance for the projection (VIP) value = 5.3, while serum urea levels and urine density higher in the MA subjects, and waist-to-height ratio, waist circumference, BMI, percentage of total body fat, body weight, and pulse pressure lower in MA adolescents appeared as variables potentially significantly contributing to between group separation, with VIP values ranging between 0.79-to-0.51 (Fig 3).

T-test comparing the variables between NA vs. MA groups confirmed the results from multivariate analyses, except for significance in pulse pressure (Table 2). Thus, microalbuminuric boys had significantly lower mean body weight, waist circumference, waist-to-height ratio, BMI, and total body fat; and higher serum urea levels and urine density (both significant within the reference range) in comparison with their normoalbuminuric counterparts.

Low prevalence of MA and a lacking relationship between ACR and CV risk factors led us to speculate whether in apparently healthy adolescents ACR in microalbuminuric range reflects cardiovascular risk factors-associated endothelial damage. It might have been an evidence of e.g. orthostatic, post-exercise, or post-infectious MA, incorrect collection (not midstream urine, lack of hygiene), recent manipulation or sexual activity. Since in apparently healthy adults albuminuria well below the threshold may be a marker for subclinical vascular damage that predisposes to future CV disease and death (reviewed in [38]), we compared the subjects presenting ACR within the upper vs. lower quartile of normoalbuminuria. In this setting high normal ACR associated with slender stature, without any significant relationship with increased cardiometabolic risk markers (Table 2). Interestingly, these slimmer boys presented more central concentration of body volume (as follows from higher ABSI) and slightly higher eGFR (within the normal range).

#### Microalbuminuria in lean vs. centrally obese boys

**Fig 4:**
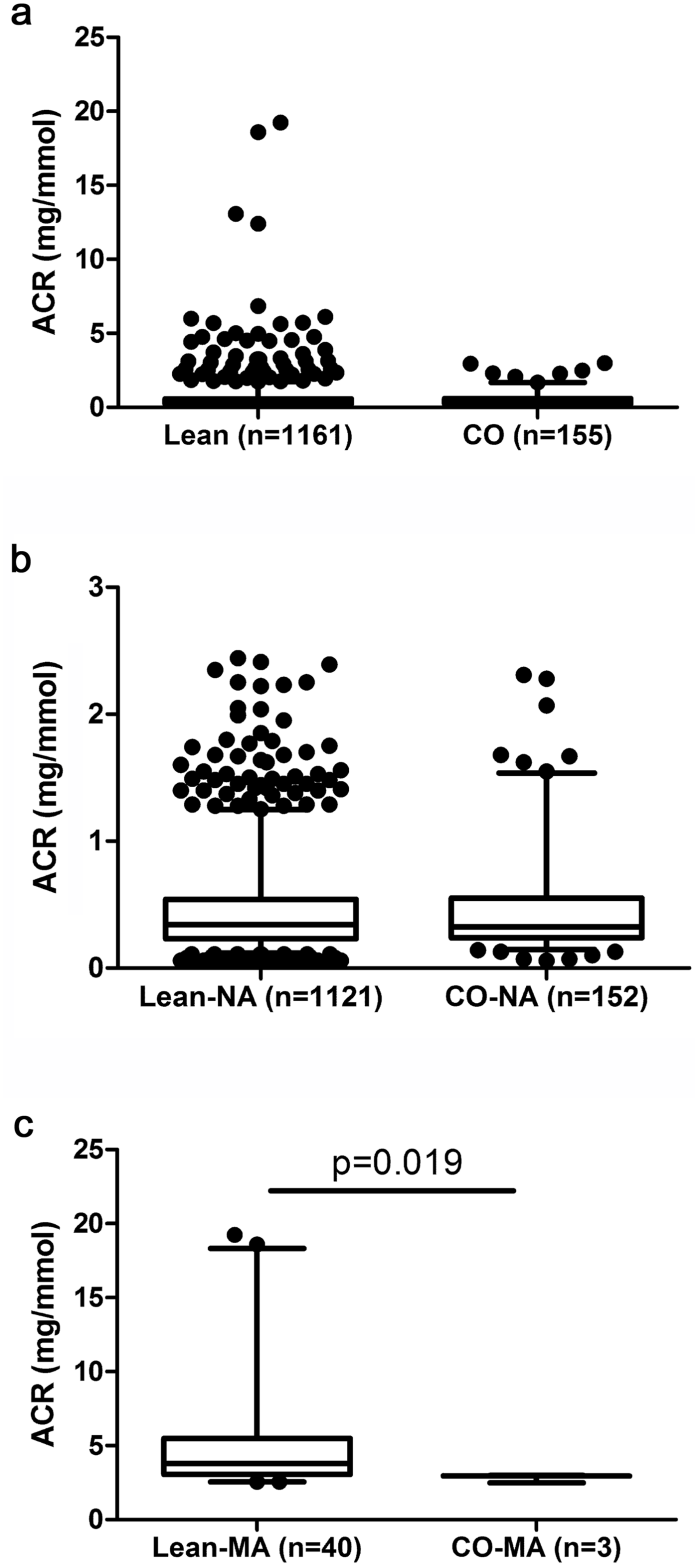
Albumin-to-creatinine ratio in lean and centrally obese boys. a/ whole cohort; b/ normoalbuminuric boys; c/ microalbuminuric boys. ACR: urinary albumin-to-creatinine ratio; CO: centrally obese subjects; box plot indicates quartiles and whiskers the 5^th^ and the 95^th^ percentiles.

Eleven point four percent of boys presented central obesity (waist-to-height ratio: 0.55±0.04 vs. 0.42±0.03, respectively; p<0.001). ACR did not differ significantly in lean versus centrally obese boys in the whole (Fig 4a), and normoalbuminuric cohorts (Fig 4b; waist-to-height ratio: 0.43±0.03 vs. 0.55±0.04, respectively; p<0.001), while centrally obese microalbuminuric boys presented significantly lower ACR if compared with their lean counterparts (Fig 4c; waist-to-height ratio 0.52±0.01 vs. 0.42±0.03, respectively; p<0.001).

Prevalence of MA in lean (3.4%) vs. centrally obese boys (1.8%) did not differ significantly (Yates’ chi-square p = 0.32).

In lean boys comparison of NA and MA groups employing OPLS-DA or t-test revealed similar results as obtained in the whole cohort. Number of centrally obese microalbuminuric boys was unfortunately too low (n = 3) to allow valid testing of associations among these subjects.

#### Girls

Similarly to the boys, OPLS-DA model showed apparent separation between NA and MA groups but explanation of variability and predictability of the model was poor (R^**2**^ = 36%, Q^**2**^ = 33%) (S1 Fig). Separation between the groups was particularly on the account of ACR (VIP = 5.4). Additional potentially significantly contributing variables to between groups separation were total body fat percentage (VIP = 0.50) lower in microalbuminuric girls, and serum albumin levels (VIP = 0.51) higher in MA girls (S2 and S3 Figs).

T-test indicated that microalbuminuric girls differ significantly from their normoalbuminuric counterparts only by lower total body fat percentage (Table 3).

Comparison between girls with NA in the upper and lower quartile confirmed that those presenting NA within the 4^th^ quartile were more slender, and presented slightly and within the reference range, but significantly, elevated fasting serum glucose, albumin, and eGRF (Table 3).

#### Microalbuminuria in lean vs. centrally obese girls

**Fig 5:**
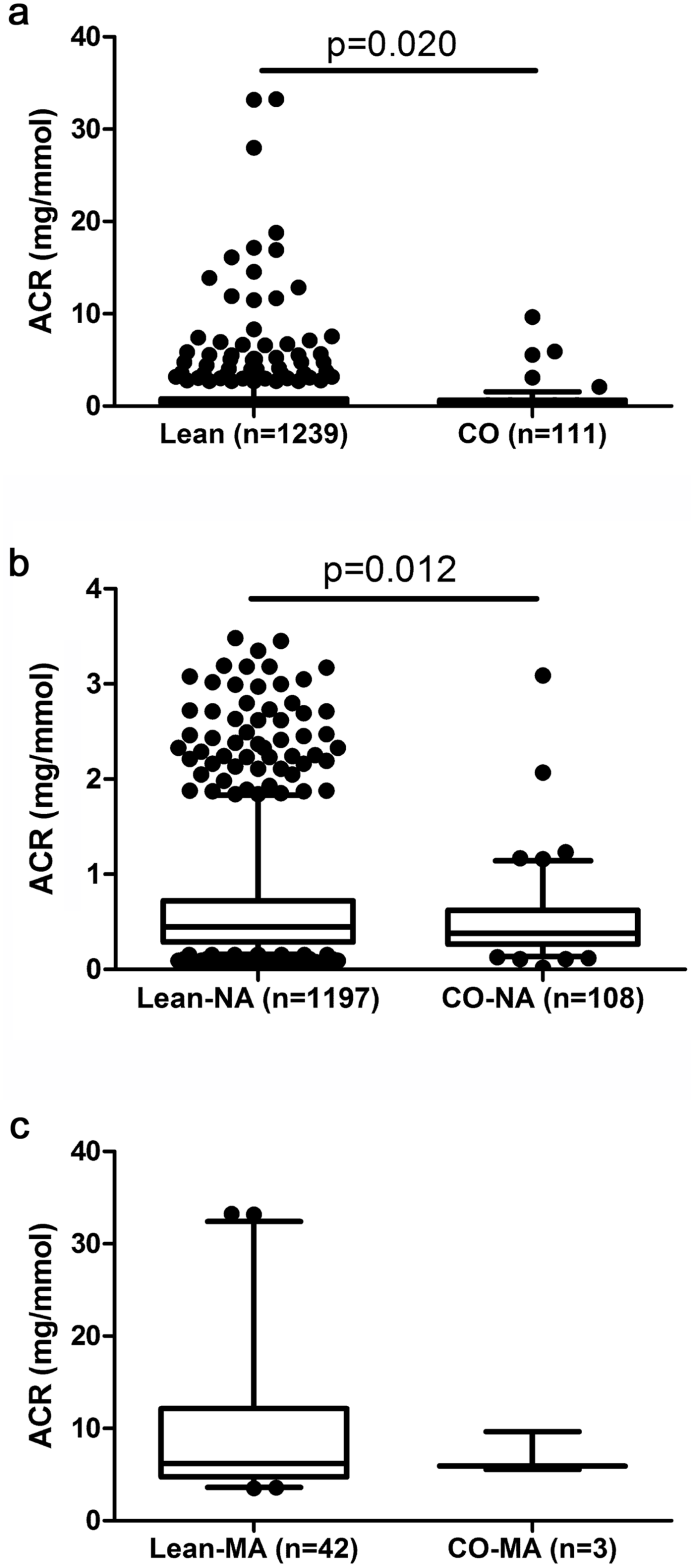
Albumin-to-creatinine ratio in lean and centrally obese girls. a/ whole cohort; b/ normoalbuminuric girls; c/ microalbuminuric girls. ACR: urinary albumin-to-creatinine ratio; CO: centrally obese subjects; box plot indicates quartiles and whiskers the 5^th^ and the 95^th^ percentiles.

Central obesity was diagnosed in 8.2% of girls. Centrally obese girls presented lower ACR if compared with the lean ones (Fig 5a; waist-to-height ratio: 0.54±0.04 vs. 0.42±0.03, respectively, p<0.001). This was on the account of lower ACR in normoalbuminuric centrally obese vs. lean girls (Fig 5b; waist-to-height ratio: 0.54±0.04 vs. 0.42±0.03, respectively; p<0.001), since ACR did not differ significantly between lean and centrally obese microalbuminuric girls (Fig 5c; waist-to-height ratio: 0.42±0.03 vs. 0.56±0.08, respectively; p<0.001).

Prevalence of MA did not differ significantly between lean (3.4%) and centrally obese (2.7%) girls (Yates’ chi-square p = 0.91).

OPLS-DA analysis as well as between group comparison using t-test yielded similar results to those obtained in the whole cohort. Since the subset of microalbuminuric centrally obese girls was too small (n = 3), further statistical analysis was not performed.

#### Comparison of microalbuminuria prevalence between the genders

Prevalence of MA was similar among lean (chi-square p = 0.94) as well as among centrally obese (chi-square p = 1.0) girls and boys.

## Discussion

Herein we present the first data on prevalence of MA in apparently healthy adolescents from Slovakia—post-communist central European country with one of the highest CV mortality rates in adults within EU-27 countries. Our study outmatches the previously published ones by number of included adolescents and particularly the number of investigated cardiometabolic markers. In contrast to our hypothesis, from the point of MA renal and thus cardiovascular health of secondary school students in Bratislava region is satisfactory: prevalence of MA is low, similar among girls and boys, regardless of presence/absence of central obesity. Mean ACR was low, although higher in girls in comparison with boys. Multivariate analysis did not reveal clear association between MA and cardiometabolic risk markers; on the contrary, MA associated with lean stature. Thus, in general population of adolescents MA is not associated with cardiometabolic risk factors.

### Prevalence of microalbuminuria

The prevalence of MA in our study (3.3%), was similar to that reported by Bangstedt et al.−3.7% [15], and lower than in the other studies on MA in general population of adolescents employing ACR determination, indicating prevalence between 8.7%-to-16.3% [9–12]. The inconsistencies might be caused due to differences in methodological approaches (timing of urine collection, analytical methods of urinary albumin assessment, different cut-off values), age, gender and racial disparities, life style related or other not completely understood factors. Our ACR analyses in the first morning spot urine sample could eliminate the falsely positive contribution of postural MA. Analysis of a single urine sample could—due to high natural intraindividual variability of UAE rate—equally bias the results of MA prevalence and levels in both directions. Our probands were not instructed to skip their usual physical activity on the day prior to biological material sampling. However, in recent study ACR did not differ significantly between students with higher and standard sport activity program [14]. Slovakia is a country with long tradition of unhealthy diet, with low intake of fruits, vegetables, fish and fiber and high intake of fats, red meat and alcohol [29]. Thus, low prevalence and low levels of MA can hardly be attributed to healthy dietary regimen, such as the consumption of Mediterranean diet [18]. Since the participation in our study was on voluntary basis we cannot exclude that we investigated an “overhealthy” cohort. Rather low prevalence of central obesity in our cohort supports this assumption.

Sanchez et al. [9] reported slightly higher prevalence of MA among adolescent boys in comparison with girls (13.5% vs. 12.9%). In other three large studies prevalence of MA was higher among girls (10.4%-to-16.3%) than boys (5.9%-to-11.0%) [7, 11, 14], while our adolescents of both genders presented similar MA prevalence (3.3%). Reason behind this disparity remains unclear. However, in accordance with several other reports our girls presented higher ACR in comparison with boys [7, 12, 14, 23, 39]. This logically stems from the lower muscle mass, and thus lower urinary creatinine excretion in females. Opposite finding in 3L study regarding that male gender was an independent significant determinant of higher ACR [18] may reflect higher incidence of benign orthostatic proteinuria among boys [40, 41].

### Association between ACR and cardiovascular risk markers

Only two studies in adolescents associated MA with standard biochemical cardiovascular risk markers, e.g. glucose homeostasis, lipid profile, or hsCRP [10, 22]. While in the large study no association between MA and biochemical variables was revealed in non-overweight subjects [10], in the smaller study a weakly significant direct relationship between UAE rate and fasting insulinemia, and surprisingly an inverse relationship to hsCRP was revealed in non-diabetic adolescents [22]. In our study only girls presenting NA within the upper quartile displayed also slightly higher fasting serum glucose concentration, not associated with increased insulinemia and insulin resistance. Thus it remains unclear whether early disturbance in glucose homeostasis associates with incipient rise in ACR. Glucose clamp techniques or at least glucose load studies, and particularly long follow-up studies are needed to elucidate this question.

Hannevold et al. reported significant direct relationship between UAE rate and systolic BP in healthy black adolescent boys, but not in black girls or white subjects of either gender [23]. Our findings correspond with those from other three studies not revealing significant association between BP and MA in apparently healthy adolescents [10, 12, 14].

Studies focusing on renal function revealed no significant association between eGRF and MA in adolescents [14, 19]. In our study subjects presenting normoalbuminuria in the upper quartile exhibited slightly elevated eGFR. This finding, together with higher serum urea levels in microalbuminuric boys and higher urine density in microalbuminuric subjects and those presenting normoalbuminuria in the upper quartile might point to higher protein intake as an additional factor contributing to increased ACR in slender adolescents. In healthy adults high chronic oral protein intake associates with high endogenous creatinine clearance correlating directly and significantly with albumin excretion rate [42]. In general population of middle-aged and elderly adults an increment of 0.1g/kg/day in protein intake increased the adjusted risk for MA significantly: OR = 1.20, 95%, CI: 1.08–1.32 [43]. Spot urine samples available in our study are not suitable for estimation of protein intake: high intake of proteins of animal origin would result in simultaneous increase in urinary excretion of urea and creatinine. To confirm our assumption on the role of higher protein intake prospective studies estimating protein intake via urea nitrogen appearance determination (requiring 24 h urine collection), and using dietary recalls are required.

### Effects of central obesity

Despite including a wide panel of potential cardiovascular risk markers and employing two different statistical approaches, we only confirmed the well known and not completely clear and understood paradox that apparently healthy adolescents presenting MA tend to be leaner than their normoalbuminuric counterparts [10, 12, 14, 15, 18–20]. This finding is generally explained as orthostatic proteinuria, a benign condition not associated with long-term risk of renal disease if isolated [13, 41]. It is attributed to a “nutcracker phenomenon“—entrapment of the left renal vein in the fork between the aorta and proximal superior mesenteric artery, which may in an upright position partially obstruct the left renal vein, leading to rise in glomerular trans-capillary hydraulic pressure difference and via the actions of angiotensin II to efferent arteriolar resistance, resulting in increased UAE [44, 45]. Thus in contrast with the adults in whom MA represents a first clinical sign of obesity-associated nephropathy [8], in otherwise healthy adolescents MA appears not to be a marker of central obesity, contrary, it associates with lean stature.

### Limitations

The strength of our study is the large number of apparently healthy adolescents investigated, analysis of numerous cardiometabolic risk markers, and a multivariate statistical approach. Particularly in specialized secondary schools of the capital and surroundings adolescents from whole Slovakia are enrolled. Thus we might speculate that our results might acceptably mirror the situation in population of white Caucasian students across the country. Our study also has several limitations. Being cross-sectional in nature it allows us to comment only on associations. Our data might be partially elusive, since based on a single urine analysis, obtained from voluntarily participating subjects. With high probability our subjects were not completely unrelated; siblings or close relatives could have participated. Low number of centrally obese microalbuminuric subjects did not allow investigating the associations in this specific cohort. Additional limitations are discussed above in relation to pertinent subject.

Taken together, our data suggest that in apparently healthy adolescents ACR is not associated with cardiometabolic risk factors. Longitudinal follow-up studies until adulthood are definitely needed to estimate the potential cardiometabolic risk of apparently healthy adolescents presenting MA, as well as to elucidate whether potential clustering of certain cardiometabolic markers predisposes apparently healthy adolescents to early development of endothelial damage reflected by MA.

## Supporting Information

S1 Fig Score scatter plot from OPLS-DA model of normoalbuminuric (NA, green circles) and microalbuminuric (MA, red circles) girls. (TIF)

S2 Fig Loading scatter plot from OPLS-DA model of normoalbuminuric (NA) and microalbuminuric (MA) girls. (TIF)

S3 Fig Plot of variables of importance contributing to between group separation among normoalbuminuric and microalbuminuric girls. Abbreviations used in S2 and S3 Figs: ACR: urinary albumin-to-creatinine ratio; WHR: waist-to-height ratio; waist: waist circumference; BMI: body mass index; uRo: urine density; weight: body weight; PP: pulse pressure; eGFR: estimated glomerular filtration rate; QUICKI: quantitative insulin check index; SBP: systolic blood pressure; Hcy: homocysteine; Crea: serum creatinine; Ins: fasting serum insulin; HDL: high density lipoprotein cholesterol; ABSI: a new body shape index; hsCRP: high sensitivity C-reactive protein; Glu: fasting serum glucose; DBP: diastolic blood pressure; Alb: serum albumin; Chol: cholesterol; UA: serum uric acid; WBC: white blood cells count; LDL: low density lipoprotein cholesterol; AIP: atherogenic index of plasma; RBC: red blood cells count; Plt: platelet count; MAP: mean arterial pressure; TAG: triacylglycerols.
(TIF)
