# Supplementary figures and images for: Presence of Cardiometabolic Risk Factors Is Not Associated with Microalbuminuria in 14-to-20-Years Old Slovak Adolescents: A Cross-Sectional, Population Study
Source: PLoS One. 2015 Jun 5;10(6):e0129311. doi: 10.1371/journal.pone.0129311 (PMC4489371; doi:10.1371/journal.pone.0129311)

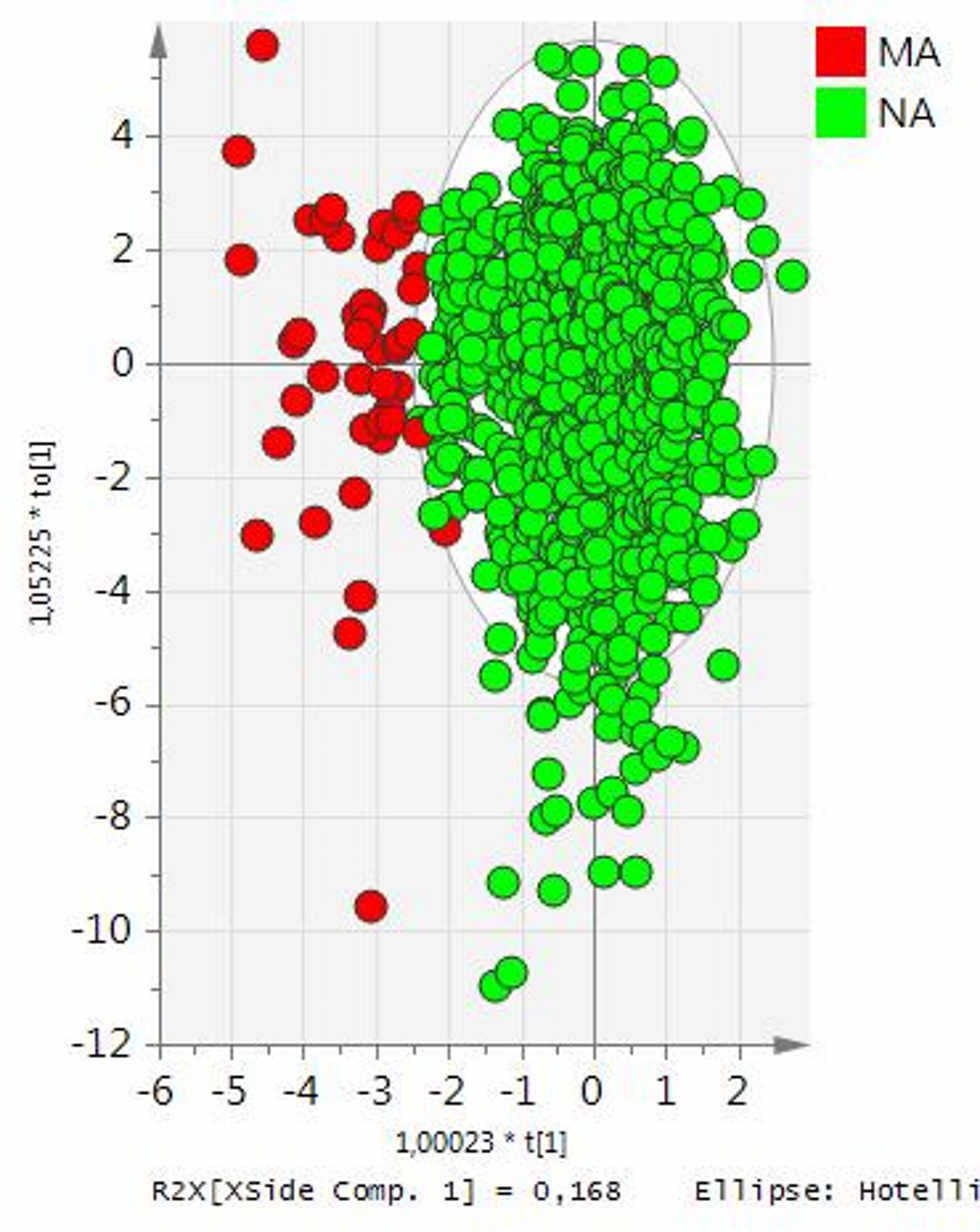

Supplement: S1 Fig — (TIF) [file pone.0129311.s001.tif]

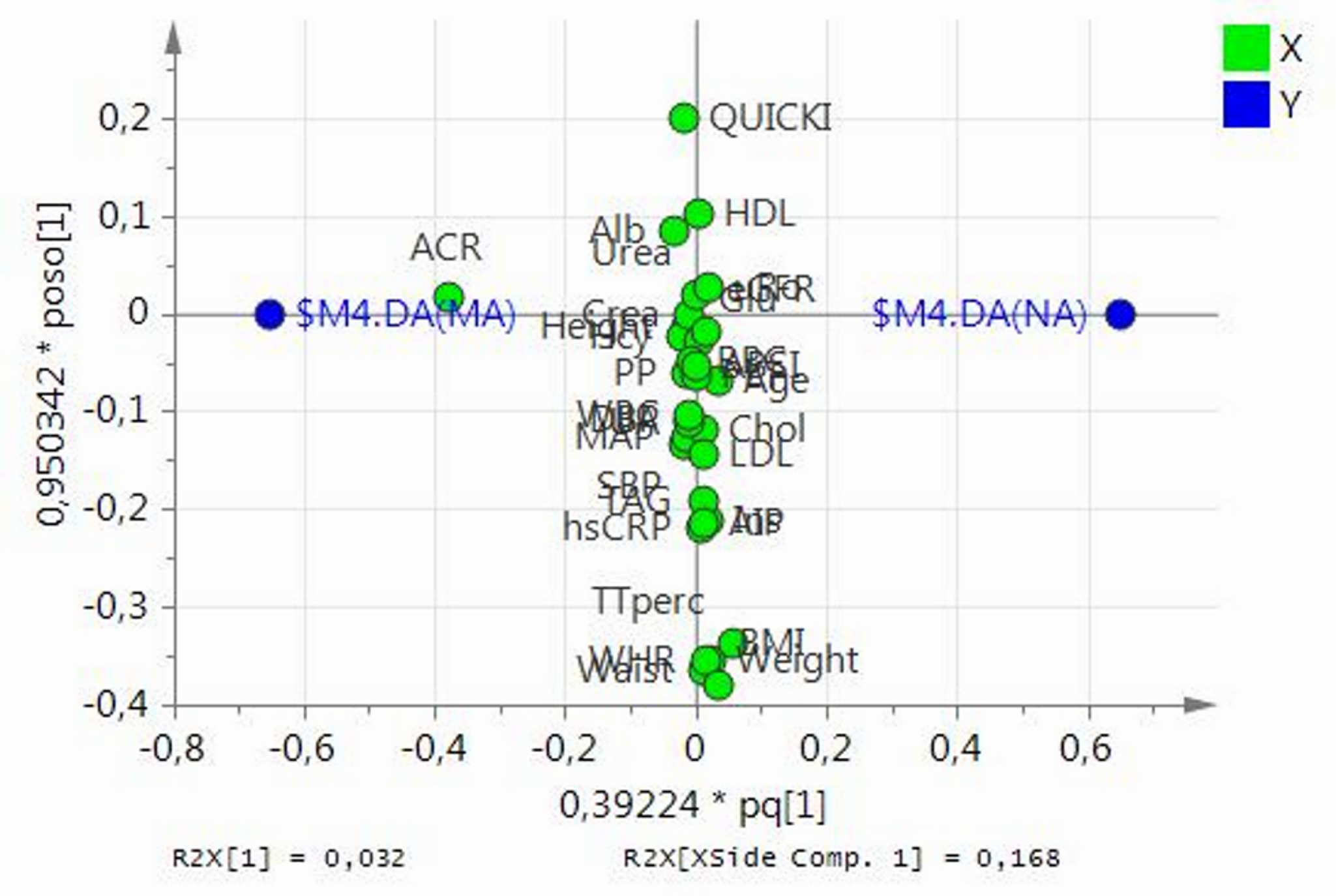

Supplement: S2 Fig — (TIF) [file pone.0129311.s002.tif]

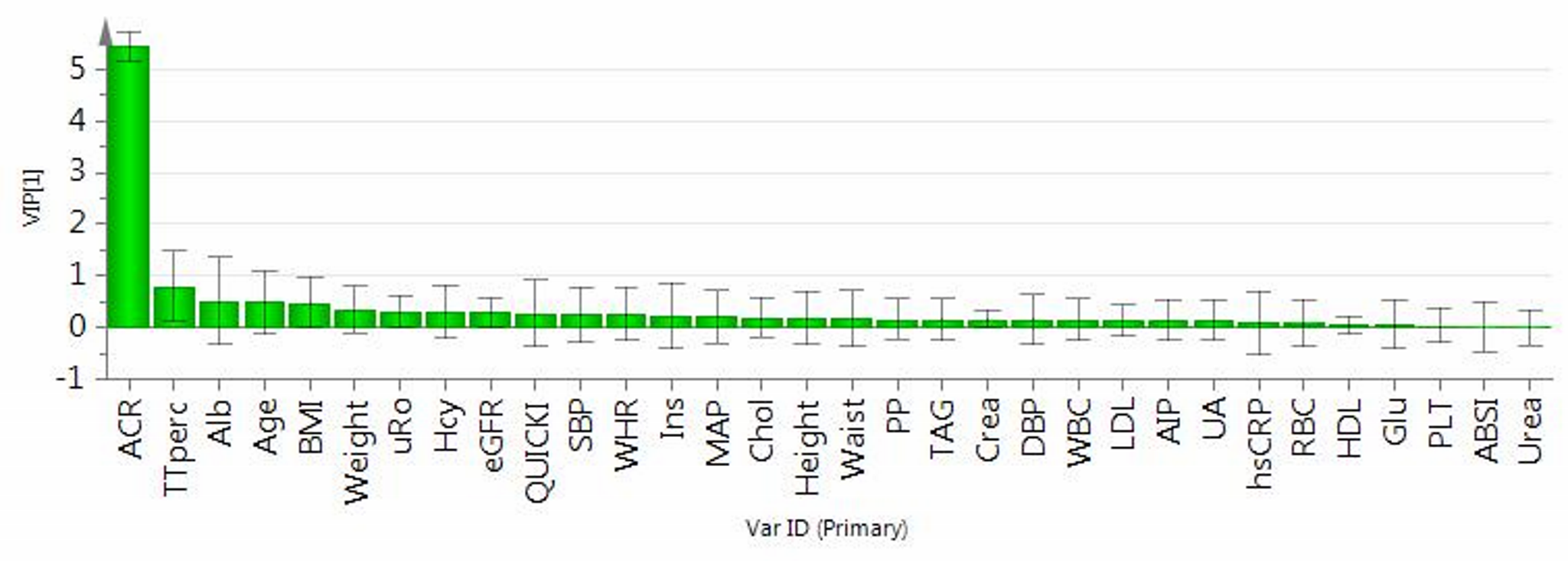

Supplement: S3 Fig — Abbreviations used in S2 and S3 Figs: ACR: urinary albumin-to-creatinine ratio; WHR: waist-to-height ratio; waist: waist circumference; BMI: body mass index; uRo: urine density; weight: body weight; PP: pulse pressure; eGFR: estimated glomerular filtration rate; QUICKI: quantitative insulin check index; SBP: systolic blood pressure; Hcy: homocysteine; Crea: serum creatinine; Ins: fasting serum insulin; HDL: high density lipoprotein cholesterol; ABSI: a new body shape index; hsCRP: high sensitivity C-reactive protein; Glu: fasting serum glucose; DBP: diastolic blood pressure; Alb: serum albumin; Chol: cholesterol; UA: serum uric acid; WBC: white blood cells count; LDL: low density lipoprotein cholesterol; AIP: atherogenic index of plasma; RBC: red blood cells count; Plt: platelet count; MAP: mean arterial pressure; TAG: triacylglycerols. (TIF) [file pone.0129311.s003.tif]
